# Technical flaws in multiple-choice questions in the access exam to medical specialties (“*examen MIR”*) in Spain (2009–2013)

**DOI:** 10.1186/s12909-016-0559-7

**Published:** 2016-02-03

**Authors:** María Cristina Rodríguez-Díez, Manuel Alegre, Nieves Díez, Leire Arbea, Marta Ferrer

**Affiliations:** Medical Education Department, University of Navarra, School of Medicine, C/Irunlarrea n°1, 31008 Pamplona, Spain

**Keywords:** Multiple choice question, Medical education, National residency examination

## Abstract

**Background:**

The main factor that determines the selection of a medical specialty in Spain after obtaining a medical degree is the MIR (“*médico interno residente”*, internal medical resident) exam. This exam consists of 235 multiple-choice questions with five options, some of which include images provided in a separate booklet. The aim of this study was to analyze the technical quality of the multiple-choice questions included in the MIR exam over the last five years.

**Methods:**

All the questions included in the exams from 2009 to 2013 were analyzed. We studied the proportion of questions including clinical vignettes, the number of items related to an image and the presence of technical flaws in the questions. For the analysis of technical flaws, we adapted the National Board of Medical Examiners (NBME) guidelines. We looked for 18 different issues included in the manual, grouped into two categories: issues related to testwiseness and issues related to irrelevant difficulties.

**Results:**

The final number of questions analyzed was 1,143. The percentage of items based on clinical vignettes increased from 50 % in 2009 to 56-58 % in the following years (2010–2013). The percentage of items based on an image increased progressively from 10 % in 2009 to 15 % in 2012 and 2013.

The percentage of items with at least one technical flaw varied between 68 and 72 %. We observed a decrease in the percentage of items with flaws related to testwiseness, from 30 % in 2009 to 20 % in 2012 and 2013. While most of these issues decreased dramatically or even disappeared (such as the imbalance in the correct option numbers), the presence of non-plausible options remained frequent.

With regard to technical flaws related to irrelevant difficulties, no improvement was observed; this is especially true with respect to negative stem questions and “hinged” questions.

**Conclusion:**

The formal quality of the MIR exam items has improved over the last five years with regard to testwiseness. A more detailed revision of the items submitted, checking systematically for the presence of technical flaws, could improve the validity and discriminatory power of the exam, without increasing its difficulty.

## Background

The examination that regulates access to medical specialties in Spain is known as the MIR exam (MIR: “*Medico interno residente*”, internal medical resident). The Ministry of Health has designed and organized this exam since 1978. The exam is officially defined as “a nationwide test in which the applicants will receive a total individual score, obtained from the sum of the result of a multiple-choice test, (carried out in the simultaneously established exam rooms in assigned locations in different regions of Spain), and the score derived from their academic merits” [1]. The aim of this exam is to objectively evaluate the applicants’ medical knowledge. Therefore, the quality of the exam is of the utmost importance. The MIR exam currently includes 225 multiple-choice questions (with 5 options), plus 10 additional backup questions to replace items excluded by the qualifying committee after the exam (due to refutations from the examinees); each error is penalized with 0.25 points. The exam score accounts for 90 % of the total score, with 10 % of the total score based on academic merits. Thus, the MIR exam is the main factor that determines the priority of the applicants for choosing the specialty and the medical center.

In order to achieve reproducibility, fairness, and validity in the exam, it is not only necessary to have questions related to a wide range of medical topics. The exam must be constructed appropriately so as to avoid possible technical flaws. The NBME defines testwiseness issues as those that “make it easier for some students to answer the question correctly, based on their test-taking skills alone”. On the other hand, irrelevant difficulties “make the question difficult for reasons unrelated to the trait that is the focus of assessment.”

Developing these questions is not an easy job because it requires the specialists who are writing them to have thorough and updated knowledge in their area of expertise, as well as to be skilled in constructing written test questions [2]. A guide for constructing written test questions has been written by the National Board of Medical Examiners (NBME), helping university teachers to improve the quality of their exams [3]. Haladyna et al. published a list of recommendations based on the reviews of scientific evidence, including studies published since 1990 [4].

The aim of this study is to analyze the technical quality of the MIR multiple-choice questions from tests given over the last five years, including both technical flaws that facilitate the answer by using testwiseness, and those related to irrelevant difficulties.

## Methods

We analyzed all the questions included in the 2009 to 2013 exams, obtained from the web page of the Ministry of Health [5].

Each exam had 235 multiple-choice questions, with five options. The study was carried out by a group of five medical doctors with different specialties as well as with expertise in constructing and analyzing multiple-choice questions.

In the first place, each question was classified depending on whether or not it included a clinical vignette and whether or not it included an image. The questions that included an epidemiological problem with actual data were considered as including a clinical vignette.

For the analysis of technical flaws, the questions were distributed among researchers based on their clinical expertise. The researchers were expert item writers with training in different medical specialties: Allergology, Neurology, Oncology, Endocrinology and Family Medicine. They had expertise in exam analysis; in less than 1 % of the questions, the researchers asked for help from other specialists (usually to confirm that an option was not plausible). At the beginning of the study, the five researchers got together to analyze a series of items in order to agree on common criteria for assessing the different issues. In addition, several meetings were held to discuss doubts that had arisen, resolving the issues by consensus among the five researchers.

We adapted the NBME guidelines for the analysis [3]. These guidelines group technical flaws into two categories: issues related to testwiseness and issues related to irrelevant difficulties as explained in the introduction.

The different issues included in the manual were grouped into the 18 categories listed in Table 1, by agreement of the five researchers. Their presence or absence was rated by means of a dichotomous scale (yes/no).

**Table 1:** Adapted questionnaire from the National Board of Medical Examiners (NBME^®^) guidelines

*Issues related to testwiseness*
The number of correct option
One or more distractors don’t follow grammatically from the stem
One or more options are collectively exhaustive
Terms such as “never” or “always” are used in options
The correct answer is longer, more specific, or more complete than other options
A word or phrase is included in the stem and in the correct answer
The correct answer includes the most elements in common with the other options
There is lack of uniformity in the options
Some of the distractors are not plausible
*Issues related to irrelevant difficulties*
The item cannot be answered without looking at the options
The answer to an item is “hinged” to the answer of a related item
Negative-phrased item (“except” or “not” in the lead-in)
Terms in the options are vague (e.g., “rarely,” “usually”)
The stem or the options are tricky or unnecessarily complicated
The stem or the options include unnecessary information
The stem or the options are too complex, with more than one concept included
Options are in an illogical order
“None of the above” or “All of the above” are used as an option
*Others*
Orthographic or syntax errors and use of outdated terms

### Statistical analysis

The data were entered into a Microsoft Excel 2010 Spreadsheet (Microsoft Corp, redmon WA, USA) and exported to STATA 12 for the analysis. We carried out a descriptive and inferential analysis. We use chi-squared tests to compare differences between years (interannual differences in the percentage of vignettes and images) and logistic regression to study tendencies throughout the five years, in the remaining analyses.

### Ethics statement

This study did not involve any personal human data.

## Results

The final number of questions analyzed was 1,143. A total of 32 questions were not included in the analysis as they had been discarded by the qualifying committee after the refutations from the examinees.

### Clinical vignettes and images

The percentage of items based on clinical vignettes increased from 50 % in 2009 to 56-58 % in the following years (2010–2013). The percentage of items based on an image increased progressively from 10 % in 2009 to 15 % in 2012 and 2013. The interannual differences in the percentage of vignettes and images were not significant.

### Technical item flaws

**Fig. 1:**
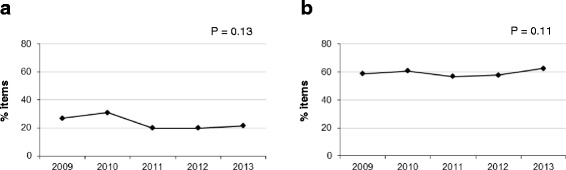
Percentage of items with technical flaws in the MIR exams from 2009 to 2013. **a**: percentage of items with technical flaws related to testwiseness. **b**: Percentage of items with technical flaws related to irrelevant difficulties

There was an overall decrease in the percentage of issues related to testwiseness throughout the five years. The main reduction (31.6 % decrease) was observed between 2010 and 2011, with stable percentages of approximately 20 % in the last years (as shown in Fig. 1a). The decrease in flawed items included most issues, but not all.

We observed no significant variation in the global percentage of issue flaws related to irrelevant difficulties in the five years analyzed, finding stable values of approximately 60 % (Fig. 1b).

### Issues related to testwiseness

**Fig. 2:**
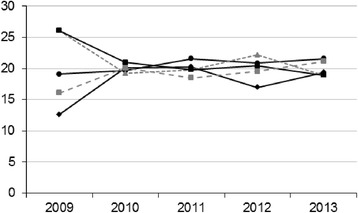
Percentage of correct answer numbers per year in the MIR exams between 2009 and 2013. Answer 1: Diamond with continuous black line. Answer 2: Circle with continuous black line. Answer 3: Triangle with discontinuous grey line. Answer 4: Square with continuous black line. Answer 5. Square with discontinuous grey line

In the first place, we observed a disproportion in the frequency of each option number in 2009 (when the most frequent correct answers were 3 and 4) that disappeared in 2010 and remained adequate in the following years (Fig. 2).

**Fig. 3:**
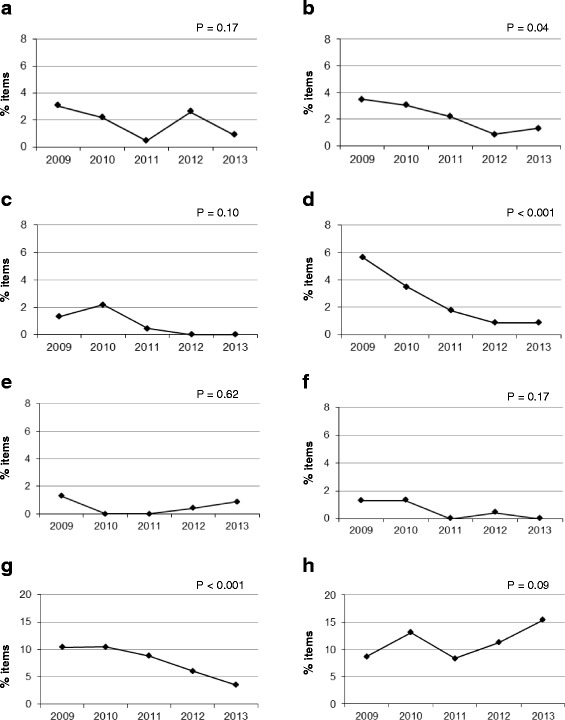
Testwiseness issues. Percentage of flawed items from 2009 to 2013. **a**: One or more distractors do not follow grammatically from the stem. **b**: One or more options are collectively exhaustive. **c**: Terms such as “never” or “always” are used in options. **d**: The correct answer is longer, more specific, or more complete than other options. **e**: A word or phrase is included in the stem and in the correct answer. **f**: The correct answer includes the most elements in common with the other options. **g**: There is lack of uniformity in the options. **h**: Some of the distractors are not plausible

Figure 3 shows the percentage of the other issues related to testwiseness in the five years analyzed. Only the issues that showed significant or near-significant changes throughout the years will be commented on in more detail.

With regard to logical clues (Fig. 3b), there is a significant decrease in the percentage of flawed items over the five years that were studied (*p* = 0.04).

In Fig. 3d, a significant improvement throughout the five years can also be observed in the “the correct answer is longer, more specific or more complete than other options” category (*p* < 0.001).

Lack of uniformity in the options was a frequent flaw in the items studied. A highly significant improvement throughout the five years that were analyzed (from 10 to 4 %) is observed in Fig. 3g (*p* < 0.001).

As shown in Fig. 3h, the most common flaw within this category was the presence of non-plausible options (10-15 % of the items). A non-significant trend (logistic regression *p* = 0.08) towards an increase in the percentage of flawed items is present.

### Item flaws related to irrelevant difficulties

**Fig. 4:**
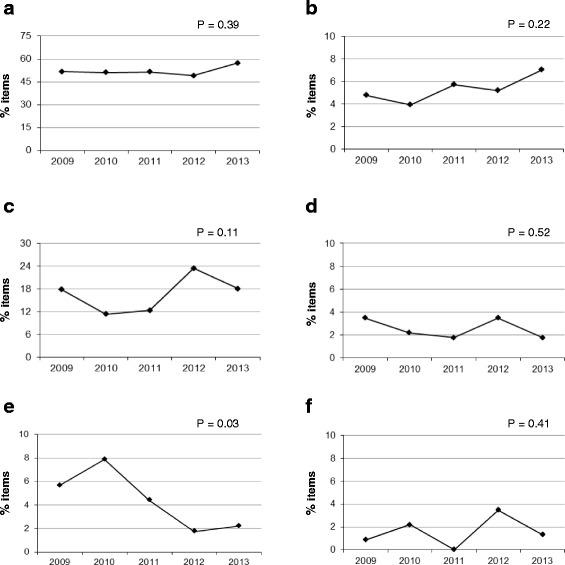
Technical flaws related to irrelevant difficulties. Percentage of flawed items in MIR exams between 2009 and 2013. **a**: The item cannot be answered without looking at the options. **b**: The answer to an item is “hinged” to the answer of a related item. **c**: Negative-phrased item (“except” or “not” in the lead-in). **d**: Terms in the options are vague (e.g., “rarely,” “usually”). **e**: The stem or the options are too complex, with more than one concept included. **f**: Options are in an illogical order

Figure 4a shows the percentage of items that cannot be answered without looking at the options, with minimal variation throughout the years. This subtype encompasses most of the flawed items found within this category (irrelevant difficulties), with percentages ranging between 50 and 57 % of the total number of items in the exam. Of note, many of these items also had other technical flaws.

Figure 4b shows a slight trend towards an increase in the percentage of items with their answer “hinged” to the answer of a related item, although the increase is not significant.

With regard to negative stems (including “except” or “not” in the lead-in), there is no improvement in the percentage of flawed items; in fact, there is actually a slight increase (Fig. 4c).

The percentage of items with stem or options that are tricky or unnecessarily complicated, with unnecessary information, or which are too complex, significantly improved; the last of these subtypes had a *p* value of 0.03 (Fig. 4e).

Within the last five years analyzed, no items were found to include the terms “none of the above” or “all of the above” within the options given.

We also looked for writing/orthographic errors and outdated terms in the items; although the percentage was quite low, there were no significant differences throughout the years (*p* = 0.76).

The maximum number of flaws found in the same question was six.

Overall, the percentage of items without any type of technical flaw (according to our analysis) did not vary significantly in the last five years (28 to 32 %).

We carried out an additional analysis comparing the percentage of flawed items in questions including and not including clinical vignettes. The results showed no differences between them (the curves overlapped), except for answers “hinged” to the answer of a previous question, which were more frequent in questions with clinical vignettes. This difference was an expected finding, as most items based on an image also include a clinical vignette, and every image always has two related questions.

## Discussion

Our study has found a high percentage of items with technical flaws in the MIR exams in the period 2009–2013. However, most of these flaws were related to irrelevant difficulties and the majority of the flaws related to testwiseness did indeed improve over the five-year period (with some of them even disappearing). The percentage of items with any type of technical flaw did not vary significantly over the last five years (68 to 72 %). According to similar studies in the literature: 28 to 75 % [6]; 35.8 to 65 % [7] and 20 % [8], this percentage falls into the high range. In the two last studies the percentage of flawed items was lower, but the number of items studied and the number of possible issues were also lower. In our study, the number of different flaws per item ranged between one and six. In the New England Journal of Medicine (NEJM) Weekly CME Program, multiple-choice questions are used for obtaining continuing medical education (CME) credits. The quality of these questions was analyzed in a recent study, in which all the questions analyzed had between 3 and 7 different types of technical flaws [9].

Technical flaws related to testwiseness favor the use of “tricks”, making it easier to answer the questions just by using test-taking abilities. Tarrant et al. observed that borderline students benefit from flawed items [6]. From our point of view, items with these flaws should be systematically rejected or amended because they could severely affect the validity of the test. Actually, most of these flaws can be avoided by following some simple rules. In our study, the main reduction (31.6 %) in the percentage of these flaws was observed in 2011, probably due to a better selection process. The most common flaw within this category, showing no improvement over the time period analyzed, was the presence of non-plausible options. Bonillo [10] analyzed the 2005 and 2006 MIR exams from a psychometric perspective, and demonstrated that one or two of the options given for several different multiple-choice questions were non-functioning. The frequency of this flaw is a good example of the difficulty involved in writing good quality multiple-choice questions. This issue could be improved by dedicating more time and effort to each multiple-choice question, or alternatively, reducing the number of possible answers, as other authors suggest [11, 12]

Irrelevant difficulties are those not associated with the subject that the question pretends to evaluate. They make the item more difficult, but they do not help to discriminate between students who are knowledgeable and those who are ignorant regarding the subject matter [6]. A more difficult question needs more time to be answered. If the mean time needed to answer each question increases, either the amount of time available for the exam has to be increased, or the number of questions has to be reduced so as to fit within the scheduled testing time. Logically, the size of this effect depends on the proportion of items with these irrelevant difficulties. This effect has little practical relevance if the percentage of flawed questions is small, but it may significantly affect the exam if the proportion is high, as in the case of this study. In fact, the number of multiple-choice questions in the MIR exam had to be reduced by 10 % (from 250 – 225, plus 10 reserve items in both cases) since 2009 due to the increased difficulty of the questions. A reduction in the number of items reduces the validity of the exam, limiting the topics that can be evaluated [3]. In addition, lower scores in the test due to increased difficulty [7] indirectly favor the weight of other components for the global score (academic merits). The percentage of weight of academic merits in the global MIR score has been reduced over the past few years. However, this weight reduction could also be indirectly achieved by simply reducing the degree of difficulty of the items in the exam.

Some subtypes of these technical flaws deserve more detailed analysis. In our study negative stems, and those that cannot be answered without looking at all the options (unfocused stem) were the most frequent mistakes; these same types of mistakes were also frequently found in other studies [6, 7]. Items with these flaws require more time to be answered. In the former case, more time is required to be able to understand what is being asked and in the latter case, the student is forced to read all the answers offered. In both of these subtypes, the options are usually longer than in non-flawed items, and frequently include different concepts thereby requiring more time to answer [4].

Percentage of questions based on a clinical vignette or on an image increased over the five-year period studied. As commented in the results section, the percentage of item flaws did not increase in the questions with a clinical vignette (with the exception of “hinged” answers). Thus, the inclusion of clinical vignettes does not decrease the technical quality of the exam. On the other hand, previous studies have indicated that clinical vignettes and images add some practical components to the knowledge evaluated in a MCQ exam, including differential diagnosis and integration of knowledge from different areas [13, 14]. Taking both facts into account, we think that the increase in the percentage of questions including clinical vignettes is globally positive.

Color images used in the exam are provided in a separate booklet. Typically, image-associated items are presented in pairs (two questions per image); this is probably done for economic reasons, as color printing is expensive. Our study shows that of the 10-15 % of items that are associated to an image, in nearly half of them (4-7 %), the answer is “hinged” to the answer of another item (presumably, the other item related to the same image). This finding implies that “hinging” (if the student does not know the answer to the first question, he/she cannot answer the second) or “cueing” (one of the questions provides clues to answer the other one) was present in most of the two-item image-related clusters. “Cueing” makes answering a question much easier for students with expertise in test-solving [6, 15]. “Hinging” gives correct credit to those students that know the answer to the first question (independently of whether they know the answer to the second one), while it prejudices those who do not know the answer to the first one, but would have known the answer to the second if they had known the answer to the first. The use of two items per image may be adequate, but the design of this type of items requires additional effort in order to avoid this kind of technical flaw. If hinging and/or cueing are not avoided, we believe that the cost/benefit use of images in the exam does not justify the inclusion of flawed questions.

A limitation of our work is the lack of individual performance data analysis to complement the technical flaw study. Modern validity theory is a unitary concept that goes beyond the technical quality of the issues [16]. This addition of might have enriched these results increasing their validity. Unfortunately, the data to evaluate individual performance was not publically available.

We agree with Palmer et al. [8] that a well-constructed multiple-choice question meets many of the educational requirements needed for this type of exam, and therefore, we believe that the MCQ format is adequate for the MIR exam. Evaluations are an essential component of learning and they influence the student’s approach to a subject. In this regard, the “MIR” exerts an influence on medical students as they shift their learning efforts towards this exam, especially in their last year of medical school [17, 18]. When writing multiple-choice questions, the same care should be taken outside the medical schools and the MIR process itself. Evaluators should conjugate their knowledge with the art of creativity when designing such questions [19].

## Conclusions

The technical quality of MIR exam items has improved throughout the last five years, especially with regard to flaws related to testwiseness, but there is still quite a bit of room for improvement. In this regard, Medical Education Units may have a fundamental role in the training of evaluators. We suggest three actions that could help improve the future quality of the MIR exam: 1) Instruct the professionals involved in writing and selecting MIR questions regarding how to avoid and detect technical flaws; 2) increase the number of questions submitted in order to have a larger pool for the selection process; 3) insist on the most frequent flaws: negative stems, items that cannot be answered without reading the options, hinged questions, and non-plausible distractors. Indeed, the ministry of Health has recently announced that the number of options will be reduced from 5 to 4 in the next MIR exam in 2016.

Some of the conclusions from our study may also be relevant beyond the MIR exam. Our results suggest that testwiseness issues are easier to eliminate than irrelevancy ones. Also, they indicate that clinical vignettes, which have many advantages in terms of evaluation, do not have a higher proportion of technical flaws. However, the use of two questions per image has a high risk of hanging or cueing flaws.
